# Assessment of the Long-Range NMR C,H Coupling of a Series of Carbazolequinone Derivatives

**DOI:** 10.3390/ijms242417450

**Published:** 2023-12-14

**Authors:** Matías Monroy-Cárdenas, José A. Gavín, Ramiro Araya-Maturana

**Affiliations:** 1 Interdisciplinary Group on Mitochondrial Targeting and Bioenergetics (MIBI), Talca 3480094, Chile; matias.monroy@utalca.cl; 2 Instituto de Química de Recursos Naturales, Universidad de Talca, Talca 3480094, Chile; 3 Instituto Universitario de Bioorgánica “Antonio González” Departamento de Química Orgánica, Universidad de La Laguna, 38206 La Laguna, Tenerife, Spain

**Keywords:** quinone, carbazolequinones, NMR spectroscopy, long-range C-H coupling, J-HMBC

## Abstract

Synthesis, the complete ^1^H- and ^13^C-NMR assignments, and the long-range C,H coupling constants (^n^*J*_C,H_) of some hydrogen-deficient carbazolequinones, assessed by a J-HMBC experiment, are reported. In these molecules, the protons, used as entry points for assignments, are separated by several bonds with non-protonated atom carbons. Therefore, the use of long-range NMR experiments for the assignment of the spectra is mandatory; we used HSQC and HMBC. On the other hand, the measured heteronuclear (C,H) coupling constants ^2^*J* to ^5^*J*) allow us to choose the value of the long-range delay used in the HMBC experiment less arbitrarily in order to visualize a desired correlation in the spectrum. The chemical shifts and the coupling constant values can be used as input for assignments in related chemical structures.

## 1. Introduction

**Figure 1:**
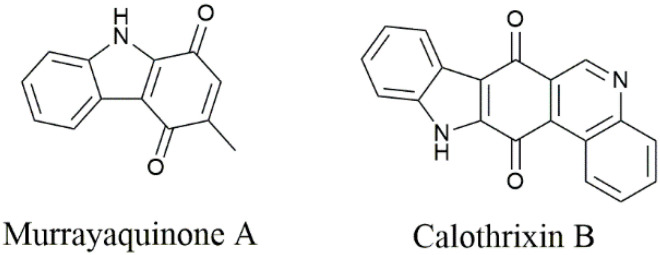
Chemical structures of Murrayaquinone A and Calothrixin B.

Molecular structure elucidation is at the basis of almost all of organic chemistry. The main tools needed to achieve this goal are nuclear magnetic resonance (NMR) spectroscopy and high-resolution mass spectrometry (HRMS). The two-dimensional (2D)-NMR experiments, based on the spin–spin coupling networks of the molecule, play a crucial role in structure elucidation [1]. Among them, heteronuclear long-range correlation experiments are essential. These experiments are the only way to connect molecular fragments through non-protonated carbons or heteroatoms. The oldest, but still the most widely used, is the HMBC experiment [2,3]. This pulse sequence consists of a few radiofrequency pulses, making it robust and the most sensitive [4]. The HMBC experiment allows structural information to be obtained using long-range correlation signals for C,H spin pairs. The detection of these correlation signals relies on the correct choice of NMR parameters, especially the long-range delay that can be adjusted while taking into account the magnitude of the coupling constants [5]. This delay is calculated as follows: Δ_2_ = 1/(2 ^n^*J*_C,H_). Since organic compounds have a range of ^n^*J*_C,H_ values, usually from 2 to 15 Hz [6], Δ_2_ should be at least equal to 100 ms. Generally, a delay shorter than the theoretical value is employed to avoid the decay of ^1^H magnetization during this delay, particularly for large molecules. When the experiment is optimized for CH-long-range couplings ^n^*J*_C,H_ in the range of 6–10 Hz, it provides access mainly to ^3^*J*_C,H_ correlations, whereas the often smaller ^2^*J*_C,H_ and ^4^*J*_C,H_ couplings generate weak or invisible correlation signals in the spectra [1,4,7]. However, the choice is generally made arbitrarily rather than from knowledge of the actual value of the couplings. Therefore, knowing the coupling constants in model molecules can help in choosing the adequate NMR parameters and, hence, correctly assigning the signals in the HMBC spectrum. This is because sometimes, four and even five-bond correlations, in addition to the common two and three-bond correlations observed using a standard value of Δ_2_ (60–80 ms), are observed. Correlations to four and even six bonds have been observed for diverse molecular structures and coupling pathways [1,6,8,9,10,11,12,13,14,15]. Some of them are as simple as ethyl crotonate, which, using a 65 ms delay [8], exhibits a five-bond correlation between the methyl protons of the alkoxy group and the Cα to the carbonyl group, a coupling pathway with a high degree of freedom. This is notable because the rigidity of the molecules has been argued as the reason we observed these non-common couplings. A special issue is the case of extremely hydrogen-deficient compounds, because their elucidation, in some cases, relies on these type of couplings. Complementary to HMBC, J-HMBC, among other NMR experiments, has been developed to visualize the very long-range connectivities [16,17,18,19,20,21,22]. The assignment of NMR spectra and measuring long-range C,H couplings provide insights into the adequate selection of parameters (mainly long-range delay in HMBC experiment) in the NMR of hydrogen-deficient molecules. These can also be used as input data for NMR calculations with theoretical methods, because they need to corroborate the accuracy of the data obtained by comparison with experimental results or combine the data to obtain information, for example, regarding conformational equilibrium in flexible molecules [23,24,25]. On the other hand, quinones have wide structural diversity [26] and are vastly distributed in nature, including in interstellar dust [27]. They have essential roles in the cell electron transport chain [28] and spark great interest in toxicology [29,30], medicinal chemistry [31,32,33], and agriculture as antifungals against phytopathogenic fungus [34,35,36]. Furthermore, quinones have relevance as dyes [37] and in energy storage, especially those with nitrogen atoms in their structures [38,39]. Among this last class of quinones, carbazole quinones have attracted interest owing to the bioactivities of some of its members [40,41]. Murrayaquinone A, among others, has shown promising cytotoxicity [41]. Calothrixin B (Figure 1) has shown a high level of in vitro cytotoxicity against the HeLa cancer cell line by interacting with human topoisomerase I and generating reactive oxygen species [42]. Some carbazolequinones show inhibitory effects on lipopolysaccharide and interferon-gamma-induced nitric oxide production in cells [43].

Our interest in biologically active quinones has led us to achieve their unequivocal structural characterization using NMR [44], assess the antitumor activity of isoquinolinequinones [45], and study carbazolequinones via mass spectrometry [46]. This work aims to study some hydrogen-deficient *o*-carbonyl carbazolequinone derivatives, whose molecular skeleton has been previously studied by us [46], in addition to measuring their long-range heteronuclear coupling constants and evaluating the effects of the substituents on them.

## 2. Results

### 2.1. Synthesis

**Scheme 1:**
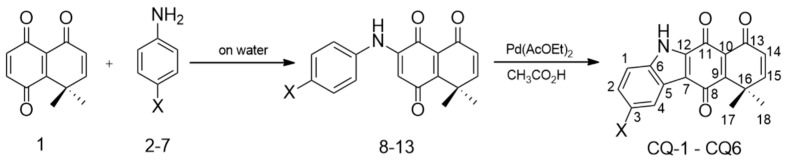
Synthesis of the studied carbazolequinones and numbering used in their structures. Compounds CQ-1 and CQ-4 have been previously described [46].

The compounds were obtained following the synthetic sequence depicted in Scheme 1, in which the first step is the on-water C-N oxidative coupling of quinone 1 with aromatic amines previously described by us [47]. In the second step, amino quinones were used as starting products in an oxidative coupling with palladium acetate under a nitrogen atmosphere, generating the corresponding *o*-carbonyl carbazolequinones CQ-1–CQ-6, following a procedure which has previously been described [46].

### 2.2. Complete NMR Assignments of the ^13^C NMR

Table 1 and Table 2 show the assignments of these spectra, which were not straightforward because the central regions of these structures consist only of carbonyl and quaternary carbon atoms aside from nitrogen atoms. The resonances of these atoms must be correlated via long-range *J*(CH) by the concerted use of ^1^H-detected one bond (C-H) heteronuclear single quantum coherence (HSQC) [48] and long-range C-H heteronuclear multiple bond connectivity (HMBC) [2].

**Figure 2:**
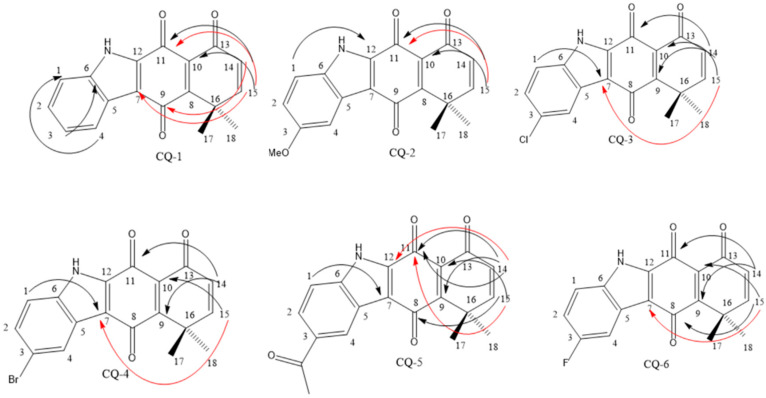
Key long-range correlations (^4^*J*_C,H_ and ^5^*J*_C,H_) observed. The red arrows show the ^5^*J* C,H coupling, and the black arrows correspond to ^4^*J* C,H coupling.

The ^1^H NMR spectra of the tricyclic skeleton of the six carbazolequinones show the protons of the AB system corresponding to the enone moiety 14-H and 15-H, where 15-H resonates at a lower field due to the resonance effect of the carbonyl group. On the other hand, the aromatic protons 4-H and 1-H are easily distinguishable from each other because of the deshielding effect that the carbonyl group at C8 exerts over 4-H. In addition, for CQ-2 to CQ-6, the multiplicity of the aromatic resonance also allows for assignment. Based on this, and using the HSQC experiment, the protonated carbons were identified. Then, via the HMBC method, the non-protonated carbons were assigned. Figure 2 shows some of the long-range correlations (^4^*J*_C,H_ and ^5^*J*_C,H_) that allowed both fragments to be joined.

Table 3, Table 4, Table 5, Table 6, Table 7 and Table 8 show the measured coupling constants (absolute values in Hz) for the studied carbazolequinones, obtained from J-HMBC experiments [16].

## 3. Discussion

The NMR spectra of the tricyclic skeleton of the six carbazolequinones were assigned as previously discussed. All molecules, except CQ-2, exhibited five-bond C,H coupling between H15 and C7 of 2–3 Hz, in the same range of several four-bond correlations. However, CQ-3 and CQ-6 also exhibited ^4^*J*_C,H_ of 5.7 and 4.1 Hz. The ^3^*J* had values in the broad range of 2.0 to 11 Hz, and the two bond correlations ranged between 1.8 and 5.6 Hz. This wide dispersion of the coupling constant values makes it difficult to predict a small range for the value of a constant. Therefore, the data obtained from reference compounds like these can be used as input data for assigning similar structures through experimental procedures or theoretical calculations.

## 4. Materials and Methods

NMR spectra were recorded on a Bruker Avance III 600 NMR spectrometer equipped with a 5 mm TCI cryogenic probe using 5 mm NMR tubes in dimethyl sulfoxide solutions. Chemical shifts were reported as ppm downfield from TMS for ^1^H NMR, and as relative to the central DMSO-d6 resonance (39.4 ppm) for ^13^C NMR. Melting points were uncorrected and were measured using an Electrothermal 9100 apparatus. High resolution mass spectra (HRMS) were obtained using a high-resolution magnetic trisector (EBE) mass analyzer. Commercially available starting materials and solvents were used without further purification. Silica gel 60 (70–230 mesh) and alu-Foil 60 F254 were used for column chromatography and analytical TLC, respectively.

Synthesis of Carbazolequinones: General Procedure

In a Schlenk tube, under an inert atmosphere, a mixture of one equivalent of the respective anilinoquinone and one equivalent of Pd(OAc)_2_ in glacial acetic acid was heated under reflux for 4 h, and then filtered. The filtered mixture was extracted 3 times with ethyl acetate and washed with a sodium bicarbonate solution; the organic phase was dried with anhydrous sodium sulfate and then evaporated under vacuum. Column chromatography on silica gel with hexane: ethyl acetate 1:1 as the eluent allowed us to obtain pure carbazolequinones.

10,10-dimethyl-5*H*-benzo[b]carbazole-6,7,11(10*H*)-trione (CQ-1) [46]

A total of 60 mg (0.24 mmol) of 3-anilino-8,8-dimethylnaphthalene-1,4,5(8*H*)-trione and Pd(OAc)_2_ 45 mg (0.18 mmol) in glacial acetic acid (4 mL) yielded 32 mg of CQ-1 (54% yield). ^1^H NMR (600.23 MHz, DMSO-d6) δ: 1.62 (s, 6H, 2XCH_3_), 6.26 (d, *J* = 10 Hz, 1H, 14-H), 7.01 (d, *J* = 10 Hz, 1H, 15-H), 7.35 (dd, *J*_1_ = 8.2 Hz, *J*_2_ = 7.9 Hz, 1H, 3-H), 7.42 (dd, *J*_1_ = 8.2 Hz, *J*_2_ = 7.9 Hz, 1H, 2-H), 7.56 (d, *J* = 8.2 Hz, 1H, 1 o 4-H), 8.11 (d, *J* = 7.9 Hz, 1H, 1 o 4-H), 12.90 (s, 1H). ^13^C NMR (150.93 MHz, DMSO-d6) δ: 26.68 (2XCH_3_), 114.39, 116.82, 122.41, 123.91, 124.57, 127.04, 127.12, 130.27, 135.68, 138.17, 158.60, 158.99, 178.12, 183.14, 183.65. IR(KBr): 3237, 1683, 741 cm^−1^. M.p.: 297–299 °C. HRMS (ESI) *m*/*z*: calculated for C_18_H_13_NO_3_ M^+^ 291.0895, 291.0894 was found.

2-methoxy-10,10-dimethyl-5*H*-benzo[b]carbazole-6,7,11(10*H*)-trione (CQ-2)

A total of 58 mg of 3-(4-methoxyphenylamino)-8,8-dimethylaminonaphthalene-1,4,5-(8*H*)-trione (0.18 mmol) reacted with 40 mg of palladium acetate (II) (0.24 mmol), yielding 32 mg of CQ-2 (55% Yield). ^1^H NMR (600.23 MHz, DMSO-d6) δ: 1.62 (s, 6H, 2XCH_3_), 3.85 (s, 3H, O-CH_3_), 6.25 (d, *J* = 10 Hz, 1H, 14-H), 7.00 (d, *J* = 10 Hz, 1H, 15-H), 7.06 (dd, *J*_1_ = 8.8 Hz, *J*_2_ = 2 Hz, 1H, 3-H), 7.46 (d, *J* = 9 Hz, 1H, 4-H), 7.52 (d, *J* = 2 Hz, 1H, 1-H), 12.83 (s, 1H). ^13^C NMR (150.93 MHz, DMSO-d6) δ: 26.66 (2XCH_3_), 55.86 (O-CH_3_), 102.29, 115.60, 116.40, 118.39, 124.95, 127.03, 130.42, 133.39, 135.64, 157.61, 158.60, 159.13, 177.79, 182.91, 183.73. IR(KBr): 3253, 1687, 836 cm^−1^. M.p.: 278–280 °C. HRMS (ESI) *m*/*z*: calculated for C_19_H_15_NO_4_ M^+^ 321.1001, 321.1007 was found.

2-cloro-10,10-dimethyl-5*H*-benzo[b]carbazole-6,7,11(10*H*)-trione (CQ-3)

A total of 180 mg of 3-((4-chlorophenyl)amino)-8,8-dimethylnaphthalen-1,4,5(8*H*)-trione (0,548 mmol) and 123 mg of palladium acetate (II) (0,548 mmol) allowed us to obtain 77 mg of CQ-3 (43% yield). ^1^H-NMR (600.23 MHz, DMSO-D6) δ 1.61 (s, 6H, 2XCH_3_), 6.26 (d, *J* = 10 Hz, 1H, 8-H), 7.02 (d, *J* = 10 Hz, 1H, 9-H), 7.44 (dd, *J*_1_ = 9 Hz, *J*_2_ = 2 Hz, 1H, 3-H), 7.58 (d, *J* = 9 Hz, 1H, 4-H), 8.06 (d, *J* = 2 Hz, 1H, 1-H), 13.10 (s, 1H, NH). ^13^C-NMR (150.93 MHz, DMSO-d6) δ 26.62 (2XCH_3_), 115.96, 116.19, 121.27, 124.79, 127.04, 127.25, 129.08, 130.38, 136.61, 136.69, 158.61, 158.91, 177.93, 182.96, 183.56. M.p.: 248–250 °C. HRMS (ESI) *m*/*z*: calculated for C_18_H_12_ClNO_3_ M^+^ 325.0506, 325.0495 was found.

2-bromo-10,10-dimethyl-5*H*-benzo[b]carbazole-6,7,11(10*H*)-trione (CQ-4) [46]

A total of 41 mg of 3-[(4-bromophenyl)amino]-8,8-dimethylnaphthalene-1,4,5(8*H*)-trione (0,11 mmol) and Pd(OAc)_2_ (25 mg, 0,11 mmol) yielded 17 mg of CQ-4 (39%). ^1^H NMR (600.23 MHz, DMSO-d6) δ: 1.61 (s, 6H, 2XCH_3_), 6.27 (d, *J* = 9.9 Hz, 1H, 14-H), 7.02 (d, *J* = 9.9 Hz, 1H, 15-H), 7.53 (d, *J* = 8.6 Hz, 1H, 4-H), 7.56 (dd, *J*_1_= 8.8 Hz, *J*_2_= 1.8 Hz, 1H, 3-H), 8.22 (d, *J* = 1.3 Hz, 1H, 1-H), 13.10 (s, 1H, NH). ^13^C NMR (150.93 MHz, DMSO-d6) δ 187.55, 181.64, 181.44, 178.41, 177.03, 159.62, 145.04, 144.27, 137.91, 130.74, 130.25, 128.01, 126.40, 118.03,112.43, 31.24, 27.63. IR: 3220, 1690, 817 cm^−1^. M.p.: 272–274 °C. HRMS (ESI) *m*/*z*: calculated for C_18_H_12_BrNO_3_ M^+^ 369.0001, 368.9987 was found.

2-acetyl-10,10-dimethyl-5H-benzo[b]carbazole-6,7,11(10H)-trione (CQ-5)

3-((4-acetylphenyl)amino)-8,8-dimethylnaphthalen-1,4,5(8H)-trione (139 mg, 0.415 mmol) and palladium acetate (II) (93 mg, 0.415 mmol) reacted, yielding 47 mg of CQ-5 (34% yield). ^1^H-NMR (600.23 MHz, DMSO-d6)δ 1.63 (s, 6H, 2XCH3), 2.67 (s, 3H, CH_3_CO), 6.28 (d, *J* = 10 Hz, 8-H), 7.02 (d, *J* = 10 Hz, 9-H), 7.64 (d, *J* = 9 Hz, 1H, 4-H), 8.00 (dd, *J*_1_ = 2 Hz, *J*_2_ = 9 Hz, 1H, 3-H), 8.70 (d, *J* = 2 Hz, 1H, 1-H), 13.19 (s, 1H, NH). ^13^C-NMR (150.93 MHz, DMSO-d6) δ 26.63, 27.25, 114.43, 117.45, 123.43, 123.95, 126.54, 127.05, 130.26, 133.28, 137.25, 140.45, 158.66, 158.81, 177.95, 183.23, 183.56. M.p.: 252–254 °C. HRMS (ESI) *m*/*z*: calculated for C_20_H_15_NO_4_ M^+^ 333.1001, 333.0995 was found.

2-fluoro-10,10-dimethyl-5*H*-benzo[b]carbazole-6,7,11(10*H*)-trione (CQ-6)

3-((4-fluorophenyl)amino)-8,8-dimetilnaphthalen-1,4,5(8*H*)-trione (220 mg, 0.7 mmol) reacted with palladium acetate (II) (151 mg, 0.7 mmol), of CQ-6 17 (97 mg, 45%). ^1^H-NMR (600.23 MHz, DMSO) δ 1.61 (s, 6H, 2XCH_3_), 6.26 (d, *J* = 10 Hz, 1H, 8-H), 7.01 (d, *J* = 10 Hz, 1H, 9-H), 7.31 (ddd, *J*_1_ = 9 Hz, *J*_2_ = 9 Hz, *J*_3_ = 3 Hz, 1H, 3-H), 7.59 (dd, *J*_1_ = 9 Hz, *J*_2_ = 4 Hz, 1H, 4-H), 7.77 (dd, *J*_1_ = 9 Hz, *J*_2_ = 3 Hz, 1H, 1-H), 13.05 (s, 1H, NH). ^13^C-NMR (150.93 MHz, DMSO) δ 26.63, 106.78, 106.95, 115.84, 116.01, 116.13, 116.19, 116.60, 116.64, 124.29, 124.36, 127.03, 130.42, 134.89, 137.00, 158.62, 159.00, 159.30, 160.88, 177.89, 182.89, 183.60. M.p.: 251–253 °C. HRMS (ESI) *m*/*z*: calculated for C_18_H_12_FNO_3_ M^+^ 309.0801, 309.0790 was found.

## Figures and Tables

**Table 1:** ^13^C assignments of the studied compounds.

Compound	CQ-1	CQ-2	CQ-3	CQ-4	CQ-5	CQ-6
X	H	OMe	Cl	Br	COMe	F
C1	114.4	115.6	116.2	116.5	114.4	116.1
C2	127.1	118.4	127.2	129.8	126.5	115.9
C3	124.6	157.6	129.1	117.1	133.3	160.1
C4	122.4	102.3	121.3	124.4	123.9	106.8
C5	123.9	123.9	124.8	125.4	123.4	124.4
C6	138.2	138.2	136.6	136.8	140.4	134.9
C7	116.6	116.6	116.0	115.8	117.5	116.4
C8	183.1	183.1	183.0	183.0	183.2	182.9
C9	159.0	159.0	158.9	158.9	158.8	159.0
C10	130.3	130.4	130.4	130.4	130.3	130.4
C11	178.1	177.8	177.9	177.9	177.9	177.9
C12	135.7	135.6	136.7	136.5	137.2	137.0
C13	183.6	183.7	183.5	183.5	183.5	183.6
C14	127.0	127.0	127.0	127.0	127.0	127.0
C15	158.6	158.6	158.6	158.6	158.7	158.6
C16	39.5	39.5	39.5	39.5	39.5	39.5
*gem*-Me	26.7	26.6	26.6	26.6	26.6	26.6
OMe		55.8				
MeCO					31.1	

**Table 2:** ^1^H assignments of the studied compounds.

	CQ-1	CQ-2	CQ-3	CQ-4	CQ-5	CQ-6
H1	7.56	7.46	7.58	7.53	7.64	7.59
H2	7.42	7.06	7.44	7.56	8.00	7.31
H3	7.35	-	-	-	-	-
H4	8.11	7.52	8.06	8.22	8.70	7.77
H14	6.26	6.25	6.26	6.27	6.28	6.26
H15	7.01	7.06	7.02	7.02	7.02	7.01

**Table 3:** Coupling constants for CQ-1.

H_4_	^2^*J* C_3_ 2.1	^3^*J* C_7_ 2.0	^3^*J* C_2_ 5.6	^3^*J* C_6_ 7.3	
H_1_	^3^*J* C_3_ 9.1	^4^*J* C_12_ 6.1	^4^*J* C_4_ 2.0		
H_2_	^2^*J* C_3_ 1.9	^3^*J* C_4_ 4.9	^3^*J* C_6_ 10.0		
H_15_	^2^*J* C_16_ 2.5	^3^*J* C_17/18_ 2.4	^3^*J* C_9_ 7.2	^3^*J* C_13_ 10.1	^4^*J* C_10_ 2.0
H_14_	^3^*J* C_16_ 7.5	^3^*J* C_10_ 4.1	^4^*J* C_17/18_ 2.0	^4^*J* C_11_ 2.1	*^4^J* C_11_ 2.1
H_17_/_18_	^3^*J* C_17/18_ 4.9	^2^*J* C_16_ 3.8	^4^*J* C_14_ 2.3	^3^*J* C_9_ 3.6	

**Table 4:** Coupling constants for CQ-2.

H_4_	^2^*J* C_3_ 2.9	^3^*J* C_6_ 7.4	^4^*J* C_1_ 2.0				
H_1_	^3^*J* C_5_ 2.9	^3^*J*C_3_ 11.0	^4^*J* C_4_ 2.6	^4^*J* C_7_ 5.7			
H_2_	^2^*J* C_1_ 2.0	^2^*J* C_3_ 2. 3	^3^*J* C_4_ 5.6	^3^*J* C_6_ 10.0			
H_15_	^2^*J* C_16_ 2.3	^3^*J*C_17/18_ 2.1	^3^*J* C_9_ 7.3	^3^*J* C_8_ 2.8	^3^*J*C_13_ 10.1	^4^*J* C_10_ 2.0	^5^*J* C_7_ 2.3
H_14_	^2^*J* C_13_ 2.1	^3^*J* C_16_ 7.5	^3^*J* C_10_ 4.2	^4^*J* C_17/18_ 2.1	^4^*J* C_9_ 2.2	^4^*J* C_11_ 2.5	
H_17/18_	^2^*J* C_16_ 3.3	^3^*J*C_17/18_ 4.9	^3^*J* C_15_ 3.7				

**Table 5:** Coupling constants for CQ-3.

HN	^2^*J* C_12_ 5.6	^2^*J* C_6_ 3.1	^3^*J* C_5_ 2.9	^3^*J* C_7_ 4.9			
H_4_	^3^*J* C_7_ 2.6	^3^*J* C_2_ 7.5	^3^*J* C_6_ 6.8	^4^*J* C_1_ 2.8			
H_1_	^2^*J* C_2_ 3.6	^3^*J* C_5_ 5.6					
H_2_	^2^*J* C_1_ 3.4	^3^*J* C_4_ 8.0	^3^*J* C_6_ 9.7				
H_3_	^2^*J* C_2_ 2.5	^3^ *J* C_1_ 8.2	^3^*J* C_5_ 9.1	^4^*J* C_6_ 2.5			
H_15_	^2^*J* C_16_ 2.8	^3^*J* C_17/18_ 2.1	^3^*J* C_9_ 7.1	^3^*J* C_13_ 10.5	^4^*J* C_10_ 1.8	^4^*J* C_8_ 2.4	^5^*J* C_7_ 2.3
H_14_	^2^*J* C_13_ 1.8	^3^*J* C_16_ 7.4	^3^*J* C_10_ 4.1	^4^*J*C_17/18_ 1.8	^5^*J* C_9_ 2.0	^4^*J* C_11_1.8	
H_17/18_	^2^*J* C_16_ 3.4	^3^*J* C_17/18_ 4.3	^3^*J* C_9_ 3.5	^4^*J* C_14_ 1.8			

**Table 6:** Coupling constants for CQ-4.

H_4_	^2^*J* C_3_ 3.5	^3^*J* C_7_ 2.1	^3^*J* C_2_ 4.6	^3^*J* C_6_ 8.0		
H_1_	^3^*J* C_5_ 5.3	^3^*J* C_3_ 10.0	^4^*J* C_12_ 5.8			
H_2_	^2^*J* C_3_ 2.2	^3^*J* C_4_ 4.5	^3^*J* C_6_ 9.0			
H_15_	^2^*J* C_16_ 3.0	^3^*J* C_17_/_18_ 3.2	^3^*J* C_9_ 7.5	^3^*J* C_13_ 10.0	^4^*J* C_10_ 2.7	^5^*J* C_7_ 2.1
H_14_	^2^*J* C_13_ 3.0	^3^*J* C_16_ 8.0	^3^*J* C_10_ 3.5	^4^*J*C_17/18_ 2.9	^4^*J* C_9_ 3.0	^4^*J* C_11_ 3.4
H_17_/_18_	^2^*J* C_16_ 3.1	^3^*J* C_17/18_ 3.5	^3^*J* C_9_ 3.5			

**Table 7:** Coupling constants for CQ-5.

H_4_	^3^*J* C_7_ 2.2	^3^*J* C_2_ 7.3	^3^*J* C_6_ 7.6	^3^*J* CO 3.6	^4^*J* C_1_ 2.8		
H_1_	^3^*J* C_3_ 7.0	^3^*J* C_5_ 5.3	^4^*J* CO 1.9	^5^*J* C_8_ 2.0			
H_2_	^3^*J* C_4_ 6.0	^3^*J* C_6_ 10.1	^3^*J* CO 2.8				
H_15_	^2^*J* C_16_ 2.7	^3^*J* C_17/18_ 2.1	^3^*J* C_9_ 7.2	^3^*J* C_13_ 10.4	^4^*J* C_10_ 2.0	^4^*J* C_8_ 3.0	
H_14_	^2^*J* C_13_ 1.9	^3^*J* C_16_ 7.6	^3^*J* C_10_ 3.9	^4^*J* C_17/18_ 3.0	^4^*J* C_9_ 2.0	^4^*J* C_11_ 2.1	^5^*J* C_12_ 3.0
H_17_/_18_	^2^*J* C_16_ 3.7	^3^*J* C_17/18_ 5.0	^3^*J* C_9_ 4.0				
CH_3_-CO	^2^*J* CO 6.0						

**Table 8:** Coupling constants for CQ-6.

H_4_	^2^*J* C_3_ 5.0	^2^*J* C_5_ 5.0	^3^*J* C_7_ 2.0	^3^*J* C_6_ 7.6			
H_1_	^3^*J* C_3_ 4.4	^3^*J* C_5_ 6.1	^4^*J* C_4_ 2.3				
H_2_	^2^*J* C3 4.1	^3^*J* C_6_ 10.1	^3^*J* C_4_ 3.9	^4^*J* C_5_ 4.1			
H_15_	^2^*J* C_16_ 2.2	^3^*J* C_17/18_ 2.0	^3^*J* C_9_ 7.1	^3^*J* C_13_ 10.5	^4^*J* C_10_ 2.0	^4^*J* C_8_ 2.7	^5^*J* C_7_ 2.3
H_14_	^2^*J* C_13_ 2.1	^3^*J* C_16_ 7.7	^3^*J* C_10_ 4.1	^4^*J* C_17/18_ 2.0	^4^*J* C_9_ 2.0	^4^*J* C_11_ 2.5	
H_17_/_18_	^2^*J* C_16_ 3.6	^3^*J* C_17/18_ 4.8	^3^*J* C_9_ 3.9				

## Data Availability

Data contained within the article.
